# Progress from morbidity control to elimination as a public health problem of schistosomiasis and the status of soil-transmitted helminth infection in Togo: a second impact assessment after ten rounds of mass drug administration

**DOI:** 10.1186/s13071-023-05882-2

**Published:** 2023-09-04

**Authors:** Ameyo M. Dorkenoo, Anna E. Phillips, Luke Klein, Fiali Lack, Essoham Ataba, Kossi Yakpa, Atna-Edi Tagba, Bozi-Esso Assoti, Efoe Sossou, Mawèké Tchalim, Gbati Datagni, Anders Seim, Marie Denise Milord, Yao Kassankogno

**Affiliations:** 1 National Program for the Control of NTDs, Ministry of Health, Public Hygiene and Universal Health Coverage, Lomé, Togo; 2 Department of Biological and Basic Sciences, Faculty of Health Sciences, University of Lomé, Lomé, Togo; 3 Family Health International 360, Washington, DC USA; 4 Centre Hospitalier Universitaire Sylvanus Olympio, Ministry of Health, Public Hygiene and Universal Health Coverage, Lomé, Togo; 5 Programme National de Lute Contre le Paludisme, Ministry of Health, Public Hygiene and Universal Health Coverage, Lomé, Togo; 6 Health and Development International, Lomé, Togo; 7 Health and Development International, Fjellstrand, Norway

**Keywords:** Preventive chemotherapy, Impact, Schistosomiasis, Soil-transmitted helminths, Togo

## Abstract

**Background:**

Due to the burden of schistosomiasis (SCH) and soil-transmitted helminths (STH), Togo Ministry of Health launched a program for Preventive Chemotherapy Neglected Tropical Diseases (PC-NTDs) in 2009, initiating integrated mass drug administration (MDA) the following year for the three PC-NTDs: SCH, STH and onchocerciasis. Significant reduction of infection across the country was noted in 2015 during the first impact assessment, following 5 years of high-coverage MDA implemented at the sub-district level for SCH and district level for STH. After another 5 years of effective MDA, a second survey was conducted in 2021 to re-evaluate the situation of SCH and STH.

**Methods:**

A cross-section of school-aged children was taken across ten districts of Togo. A total of 302 schools in 92 sub-districts were sampled, with 24 school-aged children per school resulting in 7248 children surveyed. Urine samples were tested by haemastix® for *Schistosoma haematobium*, with urine filtration for the presence of eggs conducted on haematuria-positive samples. Stool samples were collected in a subset of 34 sub-districts in seven out of the ten surveyed districts, where STH and *Schistosoma mansoni* endemicity was high during the 2015 impact assessment. Duplicate (two) Kato-Katz analysis was performed for each stool sample. Sociodemographic and school-level water, sanitation and hygiene information was also collected.

**Results:**

Overall, SCH prevalence was 5.90% (95% CI: 5.4–6.5), with 5.09% (95% CI: 4.64–5.67) for *S. haematobium* and 2.56% (95% CI: 1.98–3.29) for *S. mansoni*. STH prevalence was 19.7% (95% CI: 18.2–21.4), with 19.6% (95% CI: 18.1–21.3) hookworm, 0.08% (95% CI: 2.2–5.8) *Trichuris trichiura* and 0.04% (95% CI: 0.01–0.33) *Ascaris lumbricoides*. Compared to baseline, a significant reduction in both SCH (22.2% to 5.90%) and STH (29.2% t0 19.7%) prevalence was observed. Children aged 5–9 years were less infected than older peers aged 10–14 years: 4.76% vs. 7.53% (*P* < 0.01) for SCH and 17.2% vs. 23.0% (*P* < 0.01) for STH.

**Conclusions:**

After 10 years of high coverage integrated MDA, Togo has achieved low prevalence SCH infection through the sub-district MDA implementation with considerable infection heterogeneity within sub-districts. As STH infection has not reached a level where the infections are not a public health problem, the sub-district treatment strategy could also be adopted in addition to improvement of treatment coverage among preschool age children and hygiene and sanitation practices.

**Graphical Abstract:**

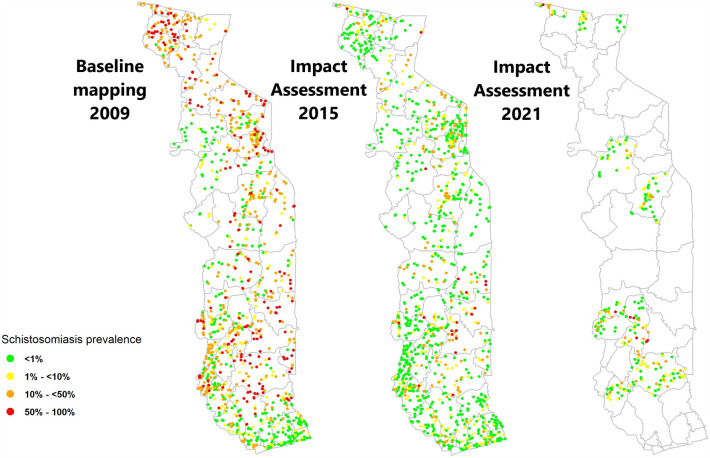

**Supplementary Information:**

The online version contains supplementary material available at 10.1186/s13071-023-05882-2.

## Background

It is estimated that over 200 million people are infected with schistosomiasis (SCH) globally and over 700 million are at risk [1] with an estimated 90% of those infected in sub-Saharan Africa [2]. Current figures suggest 2 billion people are infected with soil-transmitted helminths (STH) worldwide and a further 4 billion are at risk [2]. Both helminth infections disproportionately affect the poorest and most marginalized populations in tropical and subtropical regions where hygiene and sanitation are poor and drinking water unsafe [3].

SCH infection is caused by adult schistosomes inhabiting the human blood vessels where they excrete eggs into the bladder (for urogenital schistosomiasis caused by *Schistosoma haematobium*) or intestine (for intestinal schistosomiasis caused by *Schistosoma mansoni*) [4]. STH infection in humans is caused by ingestion of eggs (*Ascaris lumbricoides* and *Trichuris trichiura*) or penetration of the skin by larvae in the soil (hookworms *Necator americanus* and *Ancylostoma duodenale*) [5]. Routine preventive chemotherapy (PC) en masse to populations at high risk of morbidity is the recommended cost-effective public health intervention used to control morbidity caused by these infections [5].

In 2000, the Togo Ministry of Health assessed the presence of lymphatic filariasis (LF) by district [6], and after more than a decade of PC intervention in the endemic districts, the country was validated as having eliminated LF as a public health problem in 2017 [7]. Similarly onchocerciasis was mapped in Togo prior to the year 2000 followed by intermittent vector control and ivermectin mass drug administration (MDA) implemented over the years [8]. Baseline disease mapping for the three remaining PC-NTDs, SCH, STH, and trachoma, was conducted in every sub-district in all 35 districts outside the capital of Lomé between 2007 and 2009 [9]. Trachoma was not considered as a public health problem and therefore was not eligible for MDA. In 2010, Togo commenced an integrated, nationwide community-based (door-to-door) MDA against SCH, STH, and onchocerciasis. Treatment frequency and target populations were determined by the sub-district (“Peripheral Health Unit or “PHU”) prevalence for SCH, village for onchocerciasis and district level for STH, following World Health Organization (WHO) guidelines [11].

School-age children (SAC) were treated with praziquantel for SCH either annually or every other year, and with albendazole for STH either annually or twice per year, based on the sub-district and district prevalence, respectively, for the two diseases [9]. In sub-districts with SCH prevalence ≥ 50%, praziquantel is also given to adults. In addition, UNICEF conducts deworming of children aged 1–4 years during child health days in Togo. Reported programmatic treatment coverage averaged over 94% across MDA conducted between 2010 and 2014; therefore, Togo conducted an impact survey in 2015 to assess the impact of treatment. Every sub-district outside the capital was surveyed with the same schools visited at baseline and 2015 [10]. Overall prevalence declined significantly from 23.8% to 5.0% for SCH and 31.8% to 11.6% for STH [7].

Following the success of the first nationwide impact assessment in 2015, Togo embarked on a second evaluation of the status of SCH and STH in 2021 in a subset of districts that had achieved another five rounds of effective (> 75%) treatment coverage, with a view to inform the program’s next steps and, if necessary, re-categorise the treatment strategies going forward.

## Methods

### Study population

Togo had a population of > 8 million people in 2020, all of whom are at risk for STH and SCH infection [9]. At the time of baseline mapping, there were 35 of 40 districts in the country, which was restructured in 2019 to 39 districts comprising 720 sub-districts, each of which has a health centre that services 1–10 villages. Thirty-seven of the 39 districts were mapped at baseline and the same villages were re-assessed in 2015. The remaining two districts, covering the capital, Lomé, were excluded during the NTD mapping because transmission of the targeted NTDs is very low or non-existent there. To date, 638 of 720 sub-districts have been mapped. Each sub-district or PHU has a dispensary with one nurse, who provides health care to between 1 and 10 villages. In total, 92 sub-districts across ten districts (and four regions) had completed > 10 years of effective MDA against SCH and STH by 2021 and conducted a second impact assessment.

### Study design and sampling

SCH is characterised by spatial heterogeneity, and SAC constitutes the highest risk group. Considering the focal nature of SCH and the age group most affected, this survey used a cross-sectional design in a stratified, two-stage cluster survey strategy to estimate the prevalence of SCH among SAC at the sub-district level. The first selection was among villages in each sub-district, and the second selection was SAC. Since the survey aimed to estimate prevalence at the sub-district level, which is probably less ecologically heterogeneous than larger geographical areas such as districts, an estimated intra-cluster correlation coefficient (ICC) of 0.041 was used [11]. The district provided a list of villages in each sub-district. From this list, villages were selected proportional to sub-district size (that equated to approximately 30–40% of communities). In each selected village, a school was selected. If there was no school, then the neighbouring school where SAC from the selected village attended was sampled. In each school, 24 SAC (12 girls and 12 boys) aged between 5 and 14 years were sampled, which was calculated to be adequate to detect a 5% change in prevalence of SCH infection, assuming power of 80% and test size of 5%, and considering the anticipated variance in prevalence. The enrolled students were selected by class from CP1 (*cours préparatoire* I equivalent to first grade in the US) to CM2 (*cours moyen* II equivalent to sixth grade in the US) with four SAC (2 girls and 2 boys) chosen by class. To select the SAC, all students who had a signed parental consent were assembled in two lines, one for boys and one for girls. Children were selected systematically from within these two lines, using a sampling interval derived from the target sample size and the number of children.

### Sample collection and analysis

The selected schools were visited 3 days prior to data collection to explain the purpose of the survey to the school head teacher. The consent forms were then given to the SAC to give to their parent/guardian to sign and the teacher read information about the survey to the children. On the day of the survey, each selected pupil was given a container labelled with a unique barcode and requested to provide a urine sample. Urine samples were observed for macrohaematuria and tested with a dipstick (Haemastix^©^), which was read and graded within 60 s according to the manufacturer’s guideline. Only macrohaematuria and dipstick-positive urine samples (defined as trace haemolysed, +, ++, + + +) were kept for later microscopy. Urine filtration was used for egg quantification and examination [5]. Infection was categorized according to the WHO guidance where heavy infections were defined as ≥ 50 eggs per 10 ml urine [5]. Urogenital schistosomiasis infection was defined as having the presence of haematuria and/or *S. haematobium* eggs in the urine. In a subset of schools, which had any *S. mansoni* or STH infections in the last 2015 assessment, stool was examined. The stool samples were processed using the Kato-Katz technique in the available laboratories at the sub-district/PHU. Duplicate slides were done for stool samples and read for egg counts, with the average eggs across two slides taken to calculate the eggs per gram (epg) of stool for each organism. Infection of any STH was defined as the presence of at least one egg of *A. lumbricoides*, *T. trichiura*, *N. americanus* or *A. duodenale*. Thresholds for heavy-intensity infections were ≥ 50,000 epg of stool for *A. lumbricoides*,  ≥ 4000 for hookworm and ≥ 10,000 for *T. trichiura*. Infection of any schistosomiasis was defined as presence of haematuria, *S. haematobium* eggs in the urine and/or *S. mansoni* in stool (where the thresholds for heavy-intensity infections was ≥ 400 epg of stool) [12].

### Data collection and management

Data were collected between 8 November and 4 December 2021, approximately 8 months after the MDA, on Android tablets using ESPEN Collect application and cloud-based databases (https://espen.afro.who.int/tools-resources/espen-collect). Standardized forms were used to gather information about individual student demographic information and information on school-level water, sanitation and hygiene (WaSH) characteristics to help inform sustainability of SCH and STH in Togo. School- and student-level questionnaires were administered face to face, and data collectors observed the WaSH infrastructure at school. The survey team then graded water and sanitation facilities at the surveyed schools using the WHO/UNICEF Joint Monitoring Programme (JMP) for Water Supply, Sanitation and Hygiene guidelines[13]. Interviewers observed whether they were wearing shoes. School and student-level data were linked to their parasitological results using unique barcode identifiers.

Data were consolidated in Microsoft Excel (Microsoft Corp, Redmond, WA) and then analysed in R v 4.1.3 (R Core Team (2022), Vienna, Austria). Infection prevalence and average intensity of infection were calculated for schistosomiasis and STH and the 95% confidence intervals (Cis) determined using binomial and negative binomial regression models, respectively, considering clustering by schools. Infection intensities were classified into light, (moderate for STH only) and heavy infections according to WHO guidelines (Additional file 1: Table S1) [12] and the prevalence of light, moderate and heavy infections together with 95% CIs using a binomial regression model.

Association between individual- and school-level variables affecting schistosomiasis and STH infection were included in the analysis. Individual factors included age, sex, handwashing, defecation and urination, and shoe-wearing behaviours at school. School-level factors included interviewer-verified availability and type of school toilet facility and availability and type of handwashing facility equipped with water and soap. WaSH coverage was categorized using the JMP guidelines. Overall, the WaSH factors associated with STH or schistosomiasis prevalence were analysed first with univariate analysis and described as odds ratio (OR) and then mixed effects logistic regression model. All the statistical analyses were carried out using R. Graphs were developed using the ggplot2 package (v. 3.3.5) and school locations were mapped using R.

## Results

### Study population

In total, 92 PHUs were selected for the survey across 10 districts in four regions. A total of 7248 SAC were enrolled in the study and provided a urine aloe or urine and stool sample across 302 schools/villages across 10 districts in Plateaux, Centrale, Kara and Savanes regions following 5 years of MDA since the last assessment in 2015. Approximately 30 schools were surveyed in each district (range: 7–66 schools) proportional to size. Table 1 provides the number of PHU, schools, children and samples examined by district.

**Table 1:** Number of schools and children examined with mean SCH and STH prevalence by district in 2021

Region	District	Number of surveyed PHU	Number of villages/schools sampled for urine	Urine samples collected	Number of schools sampled for stool	Stool samples collected	Total number of samples	Mean prevalence of Schistosoma haematobium* % (N)	Mean prevalence of STH% (N)
Plateaux	Agou	11	26	624	15	360	624	2.56 (16)	8.33 (30)
	Akebou	3	14	336	5	120	336	10.7 (36)	61.7 (74)
	Amou	13	36	864	6	144	864	11.7 (101)	19.4 (28)
	Wawa	9	25	600	2	48	600	4.00 (24)	0
	Haho	15	66	1584	32	768	1584	4.56 (72)	25.6 (194)
Centrale	Tchaoudjo	22	56	1344	25	600	1344	3.21 (43)	18.8 (113)
Kara	Bassar	9	31	744	16	384	744	2.55 (19)	10.4 (40)
Savanes	Cinkasse	4	20	480	–	–	480	8.56 (41)	
	Kpendjal	1	7	168	–	–	168	0	
	Kpendjal Ouest	5	21	504	–	–	504	3.37 (17)	
Total		92	302	7248	101	2424	7248	5.09 (369)	19.8 (479)

^*^Presence of haematuria is the proxy of *S.haematobium*

### Prevalence of infection

**Fig. 1:**
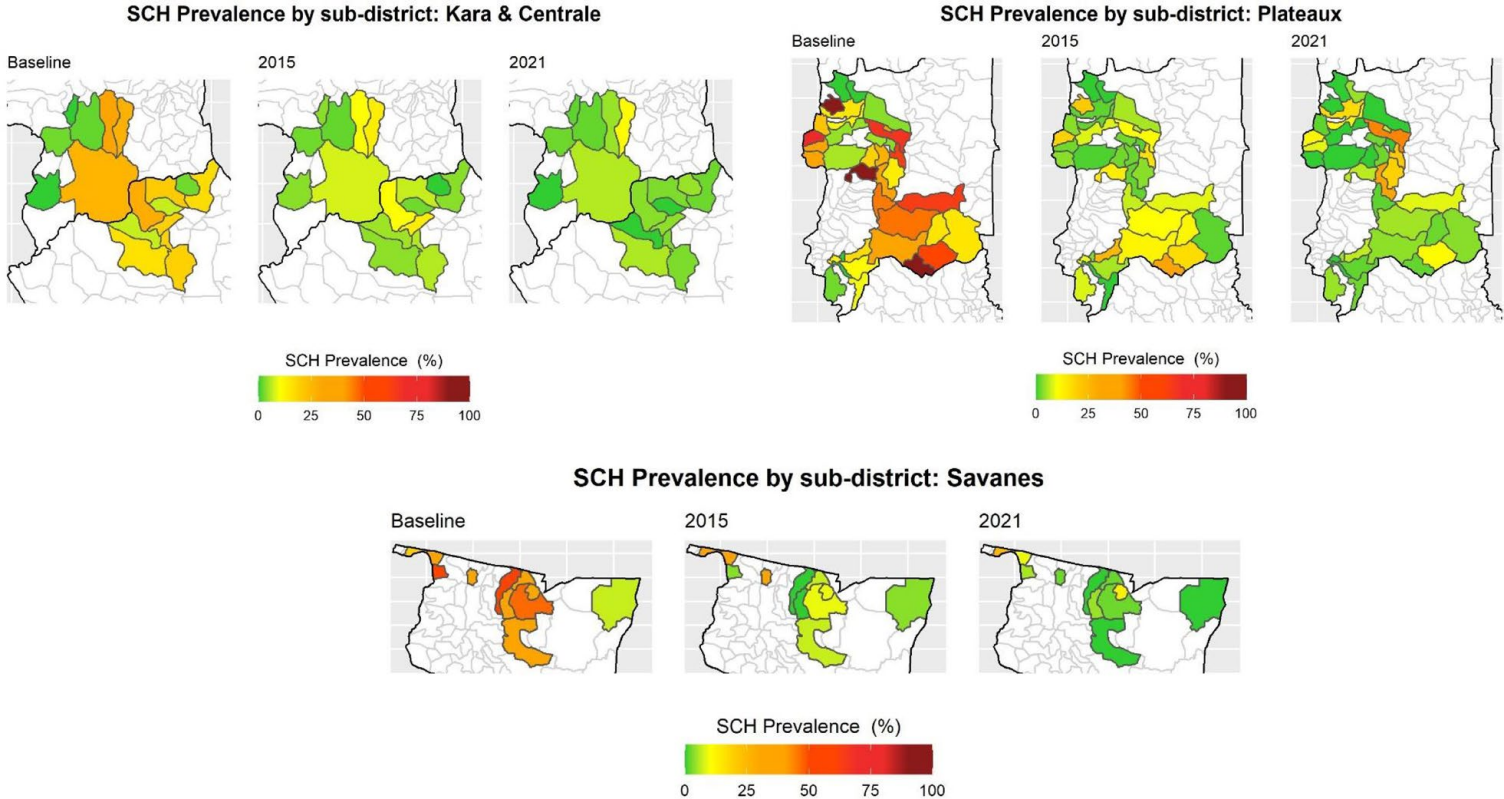
Sub-district schistosomiasis prevalence over time across four regions surveyed at all time points from 2009 to 2021 in Togo

Overall, SCH prevalence reduced significantly in the ten districts by 73.4% (*P* < 0.01) from a baseline prevalence of 22.2% to 5.90% in 2021 (95% CI: 5.4–6.5) (Table 2). The predominant SCH species in positive children was *S. haematobium* 5.09% (95% CI: 4.64–5.67), whereas *S. mansoni* was found in 2.56% (95% CI: (1.98–3.29) of the samples. Respective mean intensities of infection of 112 eggs/10 ml (95% CI: 111–113) and 13 epg (95% CI: 12.9–13.2) were seen (Table 2 and Fig. 1). *Schistosoma haematobium* showed a significant relative reduction of 73.2% (*P* < 0.01) from 19.1% as did *S. mansoni* by 38.3% (*P* < 0.01) from an initial prevalence of 4.15%.

**Table 2:** Comparison of STH and SCH prevalence in the subset of districts surveyed at baseline (2007–2009), 2015 and 2021

Survey	Any STH % (95% CI)	Hookworm % (95% CI)*	*Ascaris lumbricoides* % (95% CI)*	*Trichuris trichiura* % (95% CI)*	Any SCH % (95% CI)**	*Schistosoma mansoni* % (95% CI)*	*Schistosoma haematobium* % (95% CI)**
Prevalence % (95% CI)							
Baseline (BL)	29.2 (27.9–30.5)	28.8 (27.5–30.1)	0.21 (0.11–0.41)	0.41 (0.25–0.65)	22.2 (21.0–23.4)	4.15 (3.61–4.77)	19.13 (18–20.3)
2015	12.0 (11.1–13.0)	11.4 (10.6–12.4)	0.24 (0.13–0.44)	0.57 (0.39–0.84)	6.60 (5.90–7.30)	1.51 (1.19–1.90)	5.10 (4.51–5.76)
2021	19.7 (18.2–21.4)	19.6 (18.1–21.3)	0.04 (0.002–0.27)	0.08 (0.01–0.33)	5.90 (5.40–6.51)	2.56 (1.98–3.29)	5.09 (4.64–5.67)
RR (2015–2021)	− 64.2 (*P* < 0.01)	− 72 (*P* < 0.01)	83.1 (*P* = 0.07)	85.5 (*P* < 0.01)	10.2 (*P* = 0.13)	− 69.5 (*P* < 0.01)	− 0.55 (*P* = 0.97)
RR (BL-2021)	31.5 (*P* < 0.01)	30.8 (*P* < 0.01)	80.5 (*P* = 0.11)	79.5 (*P* = 0.02)	73.4 (*P* < 0.01)	38.3 (*P* < 0.01)	73.2 (*P* < 0.01)
Average intensity (95% CI)							
Baseline (BL)	–	6.02 (5.95–6.09)	0.09 (0.08–0.10)	0.19 (0.18–0.20)	–	–	–
2015	–	33.1 (33.6–34.0)	7.04 (6.96–7.11)	20.6 (20.5–20.8)	–	2.69 (2.64–2.73)	1.30 (1.22–1.38)
2021	–	82.5 (82.1–82.8)	0.04 (0.03–0.05)	0.15 (0.14–0.17)	–	13.0 (12.9–13.2)	112 (111–113)*
RR (2015–2021)	–	− 143.9 (*P* < 0.01)	99.4 (*P* < 0.01)	99.3 (*P* < 0.01)	–	− 401 (*P* < 0.01)	− 8510 (*P* < 0.01)*
RR (BL-2021)	–	− 1269.3 (*P* < 0.01)	55.4 (*P* < 0.01)	19.5 (*P* < 0.01)	–	–	–

*In the 2021 impact assessment, *S. haematobium* egg counts were only recorded in haematuria-positive samples and were not representative of the whole sample. In the 2015 survey, a sub-set of samples (five out of 15 SAC enrolled in the first of the two villages surveyed by sub-district) was selected for filtration

**Fig. 2:**
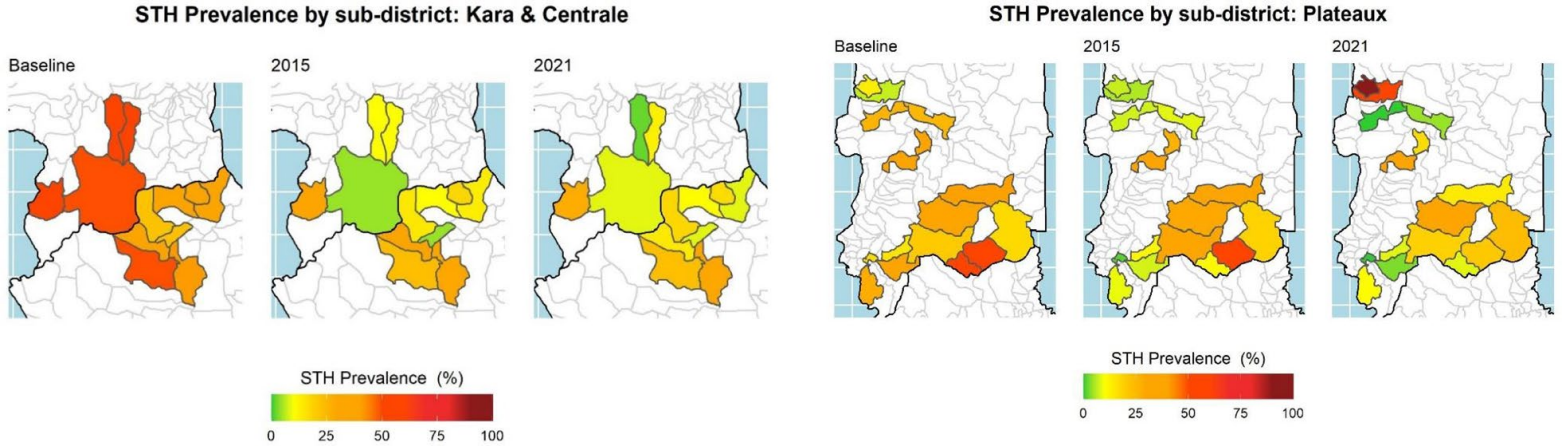
Sub-district STH prevalence over time across three regions surveyed at all time points from 2009 to 2021 in Togo

Prevalence of any STH infection (any of the three species) showed statistically significant reduction of 31.5% (*P* < 0.01), with 29.2% (95% CI: 27.9–30.5%) of children positive at baseline and 19.7% (95% CI: 18.2–21.4%) positive in 2021 (Table 2 and Fig. 2). For STH species, hookworm remained the most common STH among infected SAC in all three surveys (28.8% at baseline, 11.4% in 2015 and 19.6% in 2021), followed by *T. trichiura* (0.41%, 0.57% and 0.08%), and *A. lumbricoides* was the least common (0.21%, 0.24% and 0.04%). Hookworm and *T. trichiura* had a significant decrease in infection between baseline and 2021, with 30.8% (*P* < 0.01) and 79.5% (*P* = 0.02), respectively, but *A. lumbricoides* did not show statistically significant reductions (*P* = 0.111) due to the low numbers of infections.

**Fig. 3:**
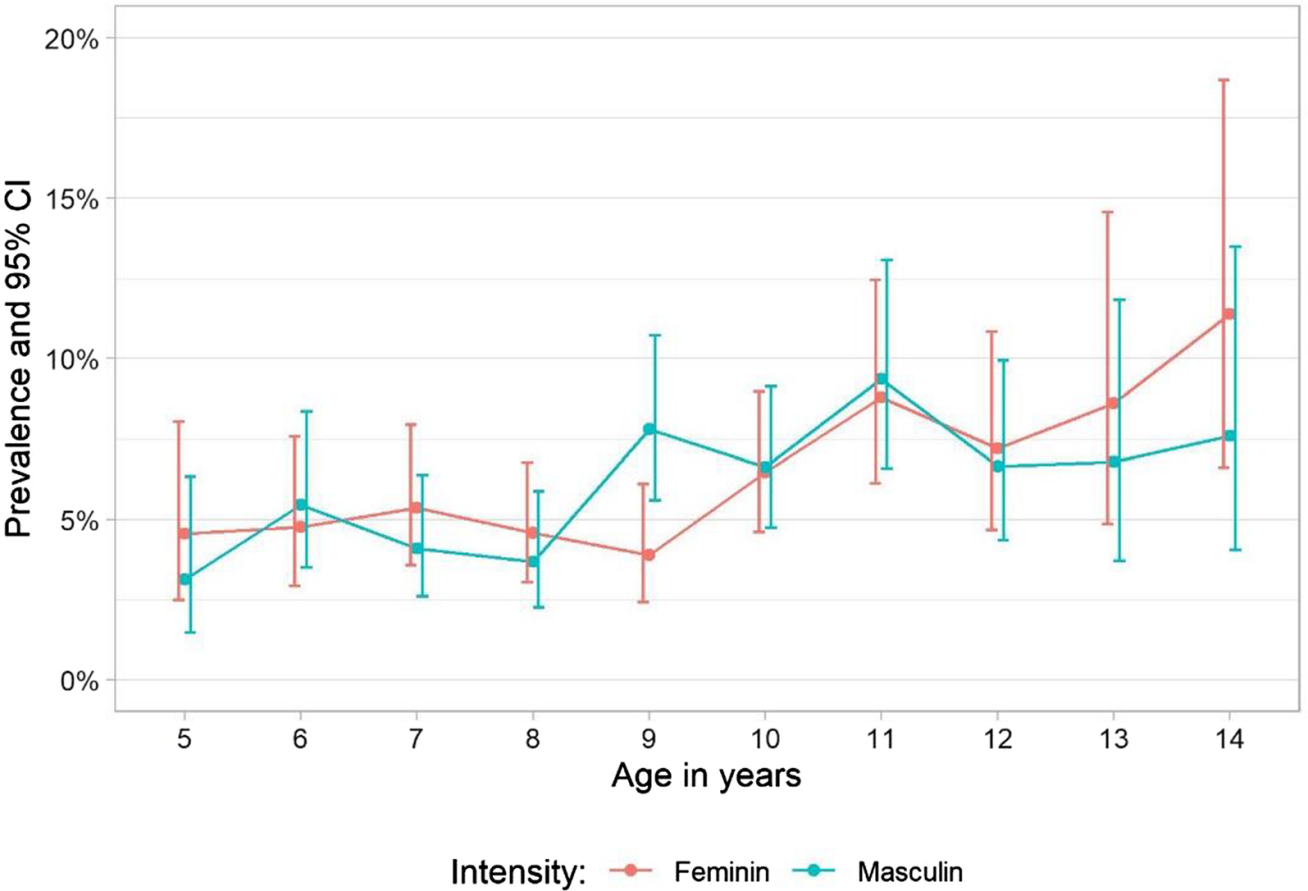
Any SCH age infection profile by gender in 2021 in Togo

Considering sex, SCH was not more common in boys than girls with an overall of 5.92% of boys positive compared with 5.91% of girls. Unlike with sex, associations with age group for SCH were statistically significant. Children aged 5–9 years were less frequently infected with SCH than their older peers aged 10–14 years (4.76% vs. 7.53%, *P* < 0.01). There was an association in the combined analysis of sex and age with schistosomiasis infection, whereby peak infection in boys occurred at 11 years old (9.36%, 95% CI 6.58–13.1%) and in girls at 14 years old (11.4%, 6.59–18.7%) (Fig. 3).

STH was more prevalent in boys with an overall of 22.7% of boys positive compared with 16.6% of girls (*P* < 0.01). In both sexes, STH infection increased with age, where SAC aged 5–9 years were less frequently infected than their older peers aged 10–14 years (17.2% vs. 23.0%, *P* < 0.01).

The intensity of STH infections was predominantly low; however, there were a few heavy-intensity SCH infections. Among SAC infected with *S. mansoni* (*N* = 62), heavy infections were found among 0.66% of the total sample. In total, 369 SAC were positive for *S. haematobium*, by either by dipstick or urine filtration. Only haematuria-positive samples were filtered (*N* = 369), of which 207 (56.3%) identified *S. haematobium* eggs and 82 (22.3%) of these had heavy infections. Of 92 PHU surveyed, 21 were above the threshold of 1% prevalence of heavy infections for elimination of *S. haematobium* as a public health problem and one of these PHU also exceeded this 1% threshold for *S. mansoni*.

### WaSH data and associations with infection

WaSH data were collected from all 302 schools and 7239 pupils during the 2021 prevalence survey (Table 3). Of these, 3164 (43.7%) had access to improved drinking water, of which 2973 (41.1%) had improved drinking water within 30 min return journey (defined as “basic” access in the JMP guidelines). There was only statistical association between basic drinking water access and *S. mansoni* (*P* < 0.01). Overall, 3570 (49.3%) of the pupils had access to improved latrines; handwashing facilities with soap and water was available to few (167 pupils, 3.79%), and water only was available to a quarter of the pupils (1103, 25.0%). There was only statistical association between improved sanitation access and reduced prevalence of *S. mansoni* (*P* < 0.01) whilst absence of handwashing facilities was associated with increased hookworm infection rates (*P* < 0.01).

**Table 3:** Relationship between hookworm and schistosomiasis infection with WaSH coverage in Togo in 2021

Characteristic	Characteristic stats	Hookworm			*Schistosoma haematobium*			*Schistosoma mansoni*			
Drinking water source in the school											
Has improved drinking water*	*N* = 7239 *n* (%)	Disease Total	Positive % (95% CI)	*P*	Disease Total	Positive % (95% CI)	*P*	Disease total		Positive % (95% CI)	*P*
	3164 (43.7)	958	18.2 (15.8, 20.8)	0.14	3164	5.47 (4.71, 6.33)	0.31	958		1.36 (0.76, 2.37)	0.02
Time < 30 min to water	*N* = 7239 *n* (%)	Disease Total	Positive % (95% CI)	*P*	Disease Total	Positive % (95% CI)	*P*	Disease total		Positive % (95% CI)	*P*
	3645 (50.4)	1152	19.7 (17.4, 22.1)	> 0.50	3645	4.75 (4.09, 5.5)	0.12	1152		4.09 (3.05, 5.44)	< 0.01
	*N* = 7239 *n* (%)	Disease Total	Positive % (95% CI)	*P*	Disease Total	Positive % (95% CI)	*P*	Disease total		Positive % (95% CI)	P
Basic	2973 (41.1)	912	18.6 (16.2, 21.4)	0.06	2973	5.48 (4.70, 6.34)	0.67	912		1.12 (0.6, 2.22)	< 0.01
Limited	191 (2.64)	48	8.33 (2.70, 20.9)		191	5.23 (2.68, 9.69)		48		4.17 (0.72, 15.4)	
Unimproved	24 (0.33)	0	–		24	4.17 (0.22, 23.1)		0		–	
Surface water	4051 (56.0)	1464	20.6 (18.6, 22.8)		4049	4.87 (4.23, 5.59)		1464		3.35 (2.51, 4.44)	
Sanitation in school											
	*N* = 7239 *n* (%)	Disease Totals	Positive % (95% CI)	*P*	Disease Total	Positive % (95% CI)	*P*	Disease total		Positive % (95% CI)	*P*
Improved	3570 (49.3)	336	16.7 (12.9, 21.2)	0.14	816	6.25 (4.73, 8.19)	0.12	336		5.36 (3.30, 8.49)	< 0.01
Unimproved	2853 (39.4)	1224	19.1 (17.0, 21.5)		3569	4.65 (3.99, 5.41)		1224		0.25 (0.06, 0.08)	
Open defecation		864	21.5 (18.9, 24.5)		2852	5.4 (4.61, 6.31)		864		4.75 (3.47, 6.44)	
Handwashing facilities at the school											
	*N* = 4410 *n* (%)	Disease Totals	Positive % (95% CI)	*P*	Disease Total	Positive % (95% CI)	*P*	Disease total		Positive % (95% CI)	*P*
Basic	167 (3.79)	48	12.5 (5.19, 25.9)	< 0.01	167	4.19 (1.85, 8.78)	0.12	48		0.03 (0, 9.23)	0.85
Limited	1103 (25.0)	144	4.86 (2.15, 10.1)		1103	6.17 (4.85, 7.79)		144		0.69 (0.04, 4.39)	
No facility	3140 (71.2)	1368	20.3 (18.2, 22.5)		3139	4.62 (3.92, 5.43)		1368		1.46 (0.92, 2.29)	

## Discussion

Overall, there has been a significant reduction in the prevalence and intensity of both SCH and STH among SAC since the start of interventions in 2010 in Togo. This can be attributed to the consistently high treatment pressure that has been applied at sub-district level, which was maintained even during the COVID-19 pandemic. The new WHO SCH recommendations encourage national control programs to shift from district-wide to sub-district MDA to avoid over- or undertreatment of areas within a district, which Togo has been doing for > 10 years [14]. The impact assessment also needed to be powered sufficiently to accurately determine the prevalence and appropriate treatment strategy for the sub-district; this resulted in a large sample size of approximately 30 villages sampled in this assessment within each district.

The global SCH prevalence in this assessment was 5.09% without a high degree of variability, and *S. haematobium* was confirmed as the predominant Schistosoma species. Although there has been a large reduction in prevalence of SCH after 10 years of treatment, relative reductions in infection appear to have plateaued in certain communities. It is important to acknowledge, however, some limitations arose from comparing baseline/2015 prevalence to the findings in 2021 due to a change in the methods used. During the 2009 baseline mapping, only dipsticks were used as a proxy for *S. haematobium* infection. During the survey in 2015, a sub-set of samples (five out of 15 SAC enrolled in the first of the two villages surveyed by sub-district) were selected for filtration. Then, in 2021, only haematuria-positive urine samples were filtered to examine egg counts as microhaematuria has been shown to be well correlated with *S. haematobium* infection [15, 16]. Haemastix® increased the number of positive children, with 162 out of 302 SAC found negative through urine filtration but positive by haemastix. This change in diagnostic strategy limited the ability to track change in intensity of urogenital schistosomiasis over time. Second, SAC aged 5 to 14 were the target sample in the 2021 impact survey, where it was found that older children aged 10–14 years were more infected with SCH and STH than younger children. The 2009 and 2015 impact survey, however, targeted SAC aged 6–9 years. Therefore, this could have limited the expected reduction in the prevalence since the 2015 impact survey.

The picture for STH is more complicated. Given that there are more species with different transmission routes and potential, there was not as clear a trend over time between the impact assessment in 2015 and 2021. Hookworm was the most common STH and *A. lumbricoides* the least common, which was also seen during the 2009 and 2015 assessments [10]. Nevertheless, there has been a modest reduction in STH prevalence after 5 years of interventions since the last nationwide impact assessment in 2015, which may be attributed to several reasons. First, MDA is implemented at the sub-district level for SCH (as opposed to the district as an implementation unit for STH), thus making it possible to target the focal aspect transmission of SCH and underpinning the new WHO guidelines [14]. Second, the 2015 SCH/STH impact survey in Togo surveyed the same schools as in 2009, but the 2021 assessment sites were randomly selected, which could have masked a reduction in prevalence in the original 2009/2015 schools. Finally, the SCH/STH assessment in 2009 and 2015 used one Kato-Katz slide for STH but in 2021 the protocol used two slides (on a single stool sample), which would have increased the sensitivity of the diagnostic. The adoption of duplicate slides in addition to a quality control reading on 10% of all samples being re-read would have increased the accuracy of STH identification particularly given the light infections in the area.

Despite these limitations, Togo has moved from its initial goal of control of morbidity to elimination as a public health problem. As expected, the initiation of PC resulted in a large reduction in both SCH and STH prevalence, and as time goes on the speed of reduction in prevalence is slowing down in certain areas and it appears a new equilibrium state has been achieved, albeit under continuing drug pressure. Currently SCH/STH support in Togo is focused on PC, yet as the country nears elimination as a public health problem and progress needs to be sustained, emphasis and efforts must also be made to improve WaSH. During this evaluation, most schools did not have adequate water and sanitation facilities, with 56% lacking improved drinking water, 51% lacking improved latrines and 71% lacking facilities for handwashing for students. Provision of clean drinking water and sanitation infrastructure has been shown to reduce helminth infection elsewhere [17] although the investment required for significant, sustained impacts on morbidity would be substantial [18]. WHO is also reinforcing snail control as part of its strategic approach to achieve the target of eliminating SCH as a public health problem and, ultimately, has the goal of elimination of transmission. There is a need for increased data on the impact of snail control in complement to mass treatment campaigns to sustain the public health impact of MDA.

Finally, hotspots for SCH transmission were identified in this assessment, which appear resilient to the PC campaigns over the past years, maintaining high prevalences and infection intensities [14, 19, 20]. It will therefore be important to intensify control interventions particularly in these communities in future years to reduce significantly and to interrupt transmission. Future surveillance of the recrudescence of the disease will need to pay special attention to ex-hotspots.

## Conclusions

The Togo Ministry of Health has made great efforts towards the development and implementation of a comprehensive NTD control program. Despite the disruptions caused by the COVID-19 pandemic, the program has adapted and should still be on target. The relatively small population and size of this country are advantages in NTD elimination. Indeed, Togo is one of the first countries to eliminate two PC-NTDs, trachoma and LF. The results of this survey show that MDA is helpful in reducing infection, but with the absence of sufficient water and sanitation services and general dependence on agrarian industry, elimination of these diseases as a public health problem or even to interrupt transmission will need additional interventions outside of MDA. Investment in WaSH infrastructure coupled with behaviour change to reduce water contact will be needed to achieve these goals. Sustainable long-term interruption of NTD transmission will depend on future collaboration with the WaSH sector, environmental change, vector control as well as strong surveillance plans. With monitoring and evaluation, Togo could serve as a model for other countries with similar epidemiological characteristics.

### Supplementary Information

**Additional file 1: Table S1.** Intensity thresholds for light, moderate and heavy infections with *Ascaris lumbricoides*, *Trichuris trichiura*, hookworms and schistosomes.

## Data Availability

The datasets used and/or analysed during the current study are available from the corresponding author, the National NTD program and the division of laboratories in the Ministry of Health in Togo upon reasonable request. As the ESPEN Collect application and cloud-based databases (https://espen.afro.who.int/tools-resources/espen-collect) were used to record the data during the survey, the database is also available from WHO-Afro. To support the electronic data, additional hard copies of these data collected are also available at the Togo's laboratoires Division.
