# Supplementary material for: Progress from morbidity control to elimination as a public health problem of schistosomiasis and the status of soil-transmitted helminth infection in Togo: a second impact assessment after ten rounds of mass drug administration
Source: Parasit Vectors. 2023 Sep 4;16:314. doi: 10.1186/s13071-023-05882-2 (PMC10478252; doi:10.1186/s13071-023-05882-2)
Supplement: Supplementary file 1 — Additional file 1: Table S1. Intensity thresholds for light, moderate and heavy infections with Ascaris lumbricoides, Trichuris trichiura, hookworms and schistosomes. [file 13071_2023_5882_MOESM1_ESM.docx]

**Additional file 1: Table S1. Intensity thresholds for light, moderate, and heavy infections with Ascaris lumbricoides, Trichuris trichiura, hookworms, and schistosomes**

| Helminth | Intensity threshold | | |
| --- | --- | --- | --- |
|  | Light | Moderate | Heavy |
| *A. lumbricoides* | 1–4999epg | 5000–49999epg | ≥50000epg |
| *T. trichiura* | 1–999epg | 1000–9999epg | ≥10000epg |
| Hookworms | 1–1999epg | 2000–3999epg | ≥4000epg |
| *S. mansoni* | 1–99epg | 100–399epg | ≥400epg |
| *S. haematobium* | 1–50 eggs/10ml urine |  | ≥50 eggs/10ml urine or visible haematuria |
| *S. japonicum* | Any intensity of infection is considered to be high | | |
